# Supplementary material for: Daunorubicin and Its Active Metabolite Pharmacokinetic Profiles in Acute Myeloid Leukaemia Patients: A Pharmacokinetic Ancillary Study of the BIG-1 Trial
Source: Pharmaceutics. 2022 Apr 5;14(4):792. doi: 10.3390/pharmaceutics14040792 (PMC9029035; doi:10.3390/pharmaceutics14040792)
Supplement: Supplementary file 1 [file pharmaceutics-14-00792-s001.zip › pharmaceutics-1650336-supplementary.pdf]

pharmaceutics

MDPI

Supplementary Materials: Daunorubicin and Its Active Metabolite Pharmacokinetic Profiles in Acute Myeloid Leukaemia Patients: A Pharmacokinetic Ancillary Study of the BIG-1 Trial

Guillaume Drevin, Marie Briet, Caroline Bazzoli, Emmanuel Gyan, Aline Schmidt, Hervé Dombret, Corentin Orvain, Aurelien Giltat, Christian Recher, Norbert Ifrah, Philippe Guardiola, Mathilde Hunault-Berger and Chadi Abbara

Supplementary Information

Table S1. Within-run and between-run reproducibility results.

		Within-run reproducibility (*n*=6)			Between-run reproducibility (*n*=6)		
Analyte	Nominal concentration (ng/ml)	Measured concentration (ng/ml)	Accuracy%	CV %	Measured concentration (ng/ml)	Accuracy%	CV %
Daunorubicin	20	20.6	103	5.11	18.3	91.6	4.04
	200	210	105	2.71	201	101	2.67
	750	746	99.5	1.4	769	103	3.48
Daunorubicinol	20	19.5	97.6	4.17	19.4	97.0	4.21
	200	206	103	2.63	203	102	3.09
	750	742	98.9	1.50	757	101	2.23

Table S2. Stability study results (n=3).

		At -20°C for 6 months		Bench-top for 12 hours		Auto-sampler for 12 hours	
Analyte	Nominal Concentration	Recovery%	CV%	Recovery%	CV%	Recovery%	CV%
Daunorubicin	20	101.33	13.40	102.67	9.61	110.33	3.84
	200	96.13	4.26	91.07	4.55	102.25	10.49
	750	96.63	4.60	99.80	5.30	91.41	5.51
Daunorubicinol	20	105.56	7.29	105.67	10.15	110.00	4.29
	200	97.73	5.50	103.53	3.77	101.33	5.12
	750	104.70	4.30	105.88	6.02	97.2	2.98

Table S3. Demographics and molecular characteristics of the 14 patients included.

Patient	Age	Sex	BSA (m2)	Cytogenetic risk group	FAB classification	CEBPA	NPM1	FLT3-ITD	FLT3-TKD	Blood blast%	Platelet (G/L)	Hb (g/dL)	Bone marrow blast%	WBC count (G/L)	CRP (mg/L)	Plasma creatinine (µmol/L)	PT %	blood lymphocytes count (G/L)
1	32	M	2.27	Adverse	M2	-	-	-	+	27	88	10.8	74	1.9	2	83	83	1.27
2	57	F	1.58	favourable	Unclassified	-	+	-	-	17	60	10.2	30	9.7	10.3	56	79	2.33
3	44	M	2.18	Adverse	M2	-	-	-	-	62.2	62.2	8.7	92	3.04	42	58	82	1.58
4	59	F	1.33	Intermediate	M5	-	+	-	-	75	34	10.3	80	118.37	30	89	53	10.65
5	52	M	1.73	Intermediate	M5	-	+	+	-	91.9	27	5.5	92	173.64	128	71	85	9.72
6	44	M	1.8	Intermediate	M2	+	-	-	0-	21.5	39	14.1	28	5.31	43	59	93	1.95
7	49	M	1.86	Intermediate	M5	-	+	+	-	18.5	68	8.1	72	2.66	179	64	71	1.79
8	58	M	1.9	Intermediate	M2	-	-	-	-	0.9	79	9.4	15	0.92	21	60	84	0.34
9	50	M	2.5	Intermediate	M5	-	+	-	-	2.5	90	7	81	64.82	299	68	68	3.89
10	45	F	1.5	Intermediate	M1	-	-	-	-	16.3	30	11.7	16.3	6.04	2	58	77	2.55

11	34	F	1.75	Intermediate	M2	-	+	+	-	66.1	75	10.8	83	9.45	109	57	87	1.58
12	60	M	2.08	Intermediate	M5	-	-	-	-	42.8	57	9.8	91	1.85	9	115	85	0.99
13	50	F	1.86	Adverse	M2	-	-	-	+	4.6	35	9.4	86	1.02	16	58	81	0.45
**14**	43	M	2.58	Adverse	M2	-	-	-	-	1.7	29	9.7	38	2.12	189	85	82	1.02

BSA: body surface area; Hb: haemoglobin; WBC: white blood cell count; CRP: C reactive protein; PT%: prothrombin time %

Table S4. Co-medications reported in the 14 studied patients.

Patients	Co-medications
1	Ondansetron - Metoclopramide
2	Insulin
3	Allopurinol - Ondansetron - Tramadol - Ceftriaxone - Amikacin
4	Allopurinol - Hydroxyurea - Paracetamol - Oxycodone - Potassium chloride - Oxazepam - Hydroxyzine - Ondansetron - Rasburicase - Furosemide - Ceftriaxone - Levofloxacin
5	Allopurinol - Hydroxyurea - Morphine - Nefopam - Vancomycin - Ceftazidime - Aciclovir - Furosemide - Insulin – Oxycodone – Oxazepam – Magnesium chloride - Ondansetron
6	Allopurinol – Paracetamol – Nefopam - Ondansetron Primperan, Heparin
7	Allopurinol - Hydroxyurea - Cefotaxime - Metoclopramide - Nefopam - Alprazolam - Valaciclovir - Paracetamol, Clarithromycin - Aprepitant - Zophren, Vancomycin - Ceftazidime - Amikacin
8	Allopurinol – Alprazolam – Posaconazole – Paracetamol - Valaciclovir - Heparin - Ondansetron
9	Allopurinol, Acupan, Furosemide, Hydrea, Magnesium, Zophren, Ceftriaxone, Ciprofloxacin
10	Allopurinol – Bromazepam – Clindamycin – Levofloxacin – Nomegestrol - Paracetamol – Zolpidem - Zophren
11	Allopurinol - Ondansetron

12	Posaconazole - Magnesium - Allopurinol - Valaciclovir - Ondansetron
13	Allopurinol – Metoclopramide – Ceftazidime – Morphine - Ondansetron
14	Posaconazole - Valaciclovir - Allopurinol - Ondansetron

Table S5. Non-compartmental analysis results.

PK parameter	Mean (Range)		Metabolic ratio (Range)
	Daunorubicin	Daunorubicinol	
AUC $_{0\text{–tlast}}$ (hr.ng/ml)	577 (375–1167)	2200 (933–4683)	0.32 (0.1–0.44)

Table S6. Optimal design for models without a covariate effect for the joint model of daunorubicin and its metabolite daunorubicinol.

	Number of elementary designs	Sampling times for each compound (hours)		Number of subjects	Total number of samples
		Daunorubicin	Daunorubicinol		
D_{opt}	5	0.5, 0.75, 1, 5, 18	0.5, 0.75, 1, 5, 18	4	200
		0.5, 1, 5, 12, 24	0.5, 1, 5, 12, 24	3	
		0.5, 0.75, 1, 10, 24	0.5, 0.75, 1, 10, 24	7	
		0.5, 0.75, 1, 5, 24	0.5, 0.75, 1, 5, 24	5	
		0.5, 0.75, 4, 10, 24	0.5, 0.75, 4, 10, 24	1	
D_{opt_one}	1	0.5, 0.75, 2, 9, 24	0.5, 0.75, 2, 9, 24	20	200

D_{opt}: optimal design, $D_{opt\ one}$: best one group design

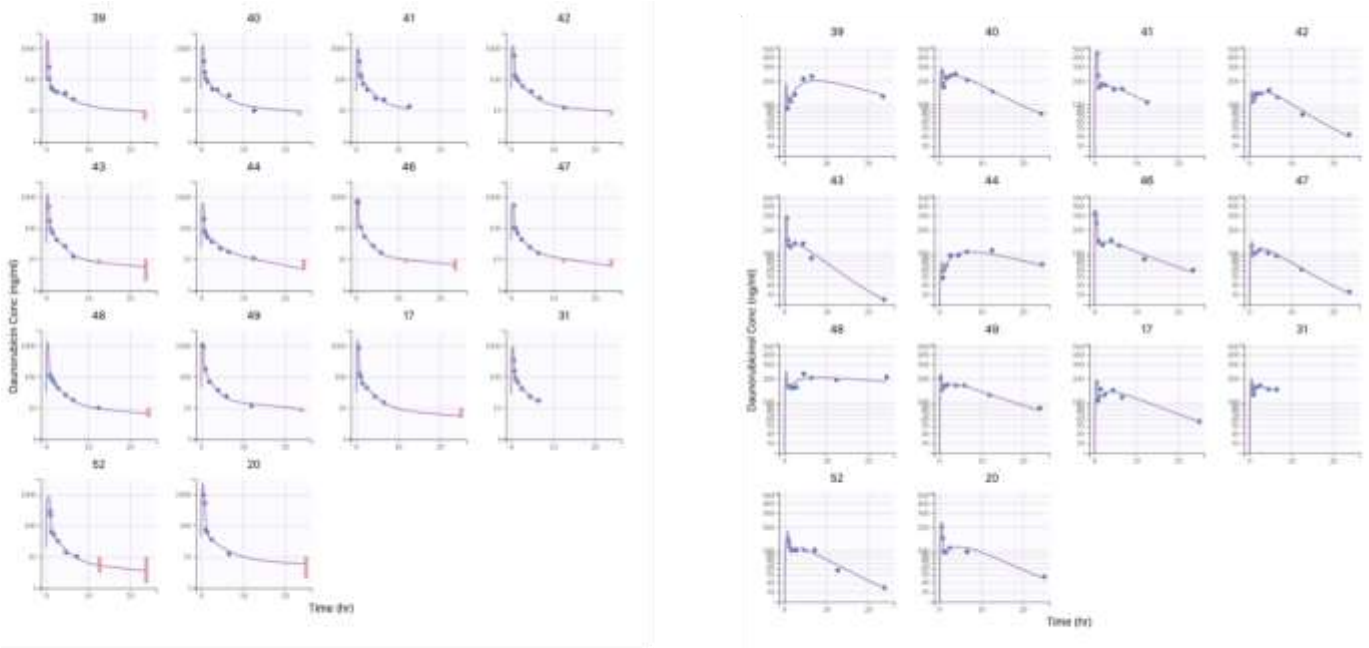

Figure S1. Predicted individual profiles fitted by means of the estimated individual final model for (A) daunorubicin and (B) daunorubicinol.

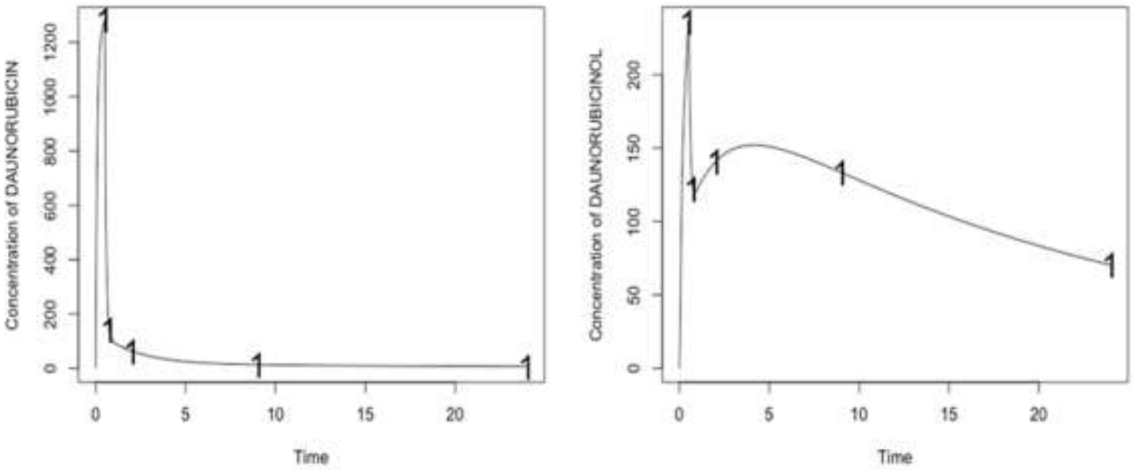

Figure S2. Concentration of Daunorubicin and Daunorubicinol versus Time for the population parameter values used in the joint parent-metabolite model. PK sampling times for D_{opt} for each compound are displayed using the number of values (i.e., 1 for one elementary design).
